# Blood management of staged bilateral total knee arthroplasty in a single hospitalization period

**DOI:** 10.1186/s13018-014-0116-1

**Published:** 2014-11-13

**Authors:** Jun Ma, ZeYu Huang, Bin Shen, FuXing Pei

**Affiliations:** Department of Orthopaedics, West China Hospital, Sichuan University, 37# Wainan Guoxue Road, Chengdu, 610041 People’s Republic of China

**Keywords:** Tranexamic acid, Multimodal blood management, Bilateral, Transfusion, Blood loss

## Abstract

**Introduction:**

Few literatures have studied the blood management in patients treated with staged bilateral primary total knee arthroplasty (TKA) in a single hospitalization period. Therefore, this study aims to evaluate the effectiveness and safety of the newly introduced multimodal blood management (MBM) in these patients.

**Materials and methods:**

We retrospectively compared the perioperative parameters in 70 cases undergoing staged bilateral primary TKA in a single hospitalization period from 2012–2013 in a single center with two different groups of patients, allocating cases to the group with the newly introduced MBM (Group A, *n* =33) and controls to the group without the newly introduced MBM (Group B, *n* =37). The newly introduced MBM protocols include preoperative hemoglobin (Hb) evaluation, high protein diet, tourniquet release after skin closure, preoperative oral iron treatment and femoral canal obturation, and one dose of tranexamic acid (TXA) IV with another one if necessary. While in the control group, only routine blood-saving techniques were used.

**Results:**

Group A had a transfusion rate of 9% (3/33), whereas 32.4% of patients (12/37) in Group B received allogenic blood transfusion. Significant benefits were also found in Group A in terms of postoperative Hb and hematocrit (Hct), reduction of postoperative pain, swelling, postoperative pain, length of stays, and hospital costs. No deep vein thrombosis (DVT) events were found in all these patients.

**Conclusions:**

The newly introduced MBM in staged bilateral TKA in a single hospitalization period can reduce blood loss effectively as well as pain and knee joint swelling instead of leading to increased complications and result in significant cost savings.

## Introduction

Total knee arthroplasty (TKA) is routinely used to treat end-staged knee diseases. It is well tolerated in the correctly selected patients, and the results are satisfactory in a high portion of them. Though the patient’s recovery is promising, yet significant intraoperative and postoperative blood loss can occur [1,2]. According to a previous study [3], the blood loss associated with unilateral primary TKA varies between 1,500 and 2,000 mL, which can reach up to as much as approximately 40% of the body’s total blood loss. This situation is worse in the patients treated with simultaneous bilateral or short-interval bilateral TKA, despite many benefits, such as reductions both in costs and length of hospital stays, better consequences of physical therapy, and earlier resolution of symptoms [4].

Thus alternative strategies, both pharmacologic and non-pharmacologic treatments, for reducing blood loss have been developed. With currently available evidences, some of the approaches, such as tranexamic acid (TXA) [5], can minimize the blood loss. Whether others, such as the use of tourniquet [6], can minimize the blood loss still remains controversial.

More and more studies report that the multimodal blood management can reduce perioperative blood loss associated with TKA effectively [7]. Since 2012, our center has conducted a newly introduced multimodal blood management (MBM). By taking this study, we want to address the following study questions: (1) How much can this newly introduced MBM minimize the perioperative blood loss and related parameters, such as transfusion rate associated with the patients treated with staged bilateral TKA during within a single hospitalization period? (2) Are there any other benefits patients can gain from the newly introduced MBM? (3) Is it safe to use the newly introduced MBM?

## Materials and methods

This retrospective study, based on data collected in our prospective database and approved by the Institutional Review Board of our institution, was performed at our center from January 2012 to December 2013 (No. 201111009). All patients, aged 18 years and older, who were scheduled for bilateral TKA in a single hospitalization period, were eligible for inclusion in the study. Exclusion criteria included revision, flexion deformity ≥30°, varus/valgus deformity ≥30°, preoperative hemoglobin (Hb) >120 g/L, contraindications for the use of TXA, and coagulation disorders. They were informed about the purpose of this study and given the written informed consent preoperatively. Each patient selected the plan that he or she preferred after the surgeon explained to them the potential advantage and disadvantage of each plan. All the surgeries were performed by one surgical team, composed of seven senior orthopedic surgeons. During this period, 72 adult patients undergoing primary staged TKA in a single hospitalization agreed to participate in this study. Two cases were dropped due to incomplete information. Two comparative groups were created: Group A, study group in which we applied the newly introduced MBM; and Group B, control group in which we applied another approach; 33 patients were allocated to Group A and 37 to Group B.

The routine perioperative blood management used in both groups includes preoperative Hb evaluation (Hb level >120 g/L) and preoperative oral iron treatment, tourniquet with 100 mmHg above systolic pressure, femoral canal obturation with bone graft, and 12-h continuous drainage.

In Group A, the surgeons adhered to the newly introduced MBM that includes a perioperative high diet, one dose of IV-administered TXA (15 mg/kg) before inflation of the tourniquet, and additional one dose of IV-administered TXA (10 mg/kg) if the drainage volume of the first 3 h is more than 300 mL.

The surgical team performed TKA in the standard way, using a midline skin incision, a standard medial parapatellar approach, and a measured resection technique. Three varieties of cemented total knee systems were used: the Scorpio NRG (Stryker Allendale, NJ, USA), PFC (Depuy, IN, USA), and Triathlon (Stryker Allendale, NJ, USA). All patients were operated under general anesthesia.

A half dose of low-molecular weight heparin (LMWH) (0.2 mL 2,000 IU) was started 6 h postoperatively and repeated at 24-h intervals with a full dose (0.4 mL 4,000 IU) in the subsequent days if there was no contraindication. Besides, an intermittent foot sole pump system was used as a routine practice to prevent deep vein thrombosis (DVT). After the discharge, 10 mg rivaroxaban was administered orally to the patients for 10 days if no bleeding events happened. Doppler ultrasound was used to diagnose the deep vein thrombosis on postoperative day 1 (POD1), postoperative day 3 (POD3), the time of discharge, and 1 month postoperatively.

To ensure the similar cohorts, we calculated the age at the time of surgery, body mass index (BMI), American Society of Anesthesiologists (ASA) grade, knee range of motion (ROM), preoperative visual analog scale (VAS), and preoperative Hospital for Special Surgery (HSS) knee scores. The preoperative knee circumferences at both the upper pole of the patella and the lower pole of the patella were measured to ensure similar patient body habitus. Measurements were taken when the patients were placed in their knee extension position.

We made a comparison in terms of operative time, tourniquet time, estimated blood loss, the length of hospital stays, drainage volume, transfusion requirements, and hospital costs between the two groups. As the primary outcomes of this study, Hb and hematocrits (Hct) were measured immediately preoperatively, immediately postoperatively, and on postoperative days 1, 2, and 3. Estimated blood loss was calculated using the modified Gross formula. The criterion for a blood transfusion was set as an Hb level of <70 g/L or 70 g/L ~100 g/L with symptomatic anemia.

Function was assessed by means of HSS. The HSS score was got at the time of charge and discharge to find out the different functional outcomes between groups. Perioperative VAS pain scores were obtained by a physician assistant blinded to the type of blood management. All clinical data were compiled and collected by a separate research associate.

All data management and statistical analysis were performed with SPSS version 18.0 software (SPSS Inc., Chicago, IL, USA). The Student *t* test and Fisher exact test were used to compare data between the two groups. Parameters that were measured in the same group but at different times were analyzed with the repeated measures general linear model. The level of significance was set at *p* <0.05.

## Results

### Blood loss

**Figure 1:**
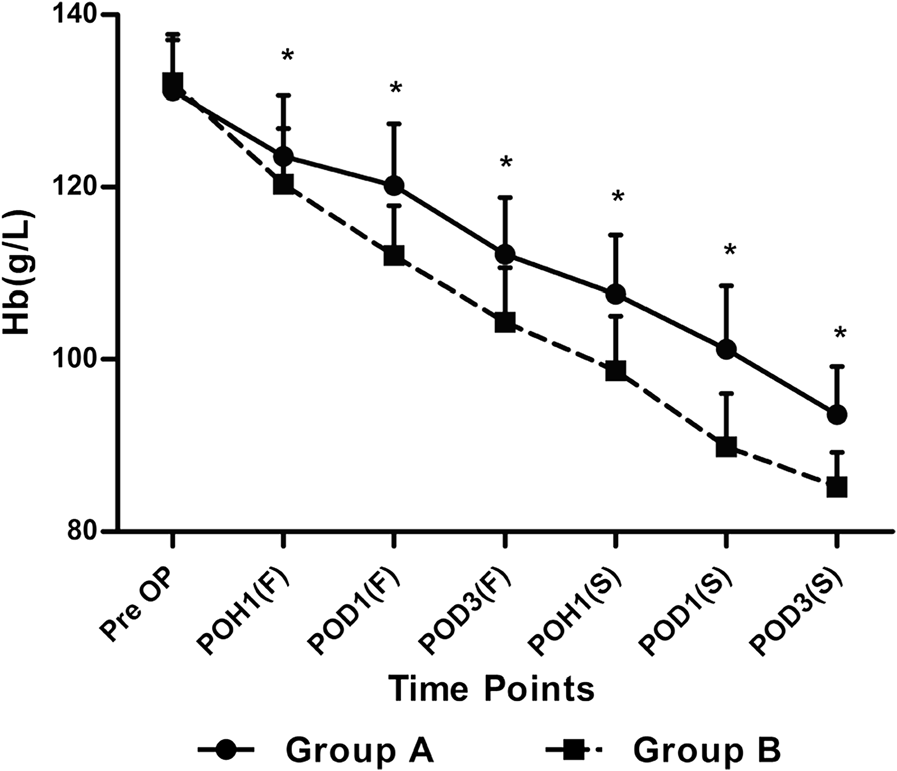
**The perioperative levels of hemoglobin (Hb) at each time point.** *Pre OP* preoperative, *POH1(F)* 1st hour after the first surgery, *POD1(F)* 1st day after the first surgery, *POD3(F)* 3rd day after the first surgery, *POH1(S)* 1st hour after the second surgery, *POD1(S)* 1st day after the second surgery, *POD3(S)* 3rd day after the second surgery. The asterisks indicate values that were significantly different between the groups.

**Figure 2:**
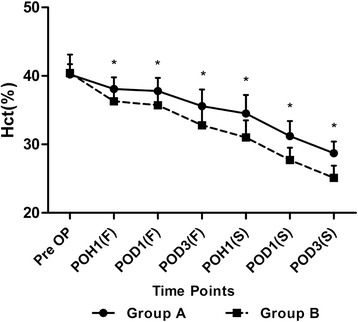
**The perioperative levels of hematocrits (Hct) at each time point.** *Pre OP* preoperative, *POH1(F)* 1st hour after the first surgery, *POD1(F)* 1st day after the first surgery, *POD3(F)* 3rd day after the first surgery, *POH1(S)* 1st hour after the second surgery, *POD1(S)* 1st day after the second surgery, *POD3(S)* 3rd day after the second surgery. The asterisks indicate values that were significantly different between the groups.

The patient demographics and clinical data were comparable between the two groups, as shown in Table 1. Both Hb and Hct levels in the two groups showed significant decreases postoperatively, and they both reached the lowest point on the postoperative day 3 of each surgery (Figures 1 and 2). The mean levels of Hb and Hct showed significant advantages in Group A at each time points postoperatively, compared with those in Group B, that is 123.6 ± 7.0 VS 120.3 ± 6.5 (*p* =0.04); 37.8 ± 1.7% VS 35.7 ± 1.9% (*p* <0.001) at 1 h after the first surgery (POH1(F)), 120.2 ± 7.2 VS 112.5 ± 5.8 (*p* <0.001); 37.8 ± 1.9% VS 35.7 ± 2.1% (*p* <0.001) on the 1st day after the first surgery (POD1(F)), 112.2 ± 6.6 VS 104.3 ± 6.3 (*p* <0.001); 35.6 ± 2.4% VS 32.8 ± 2.5% (*p* <0.001) on the 3rd day after the first surgery POD3(F), 107.6 ± 6.8 VS 98.7 ± 6.3 (*p* <0.001); 34.5 ± 2.7% VS 31.0 ± 2.5% (*p* >0.001) at 1 h after the second surgery (POH1(S)), 101.2 ± 7.3 VS 89.8 ± 6.2 (*p* <0.001); 31.2 ± 2.2% VS 27.7 ± 1.8% (*p* <0.001) on the 1st day after the second surgery (POD1(S)), 93.6 ± 5.6 VS 85.2 ± 4.0 (*p* <0.001); 28.7 ± 1.7% VS 25.1 ± 1.8% (*p* <0.001) on the 3rd day after the second surgery (POD3(S)) (Figures 1 and 2).

**Table 1:** **Preoperative demographics**

**Demographic**	**Group A (** ***n*** **=33)**	**Group B (** ***n*** **=37)**	***p*** **value**
Age* (year)	62.7 (10.4)	62.0 (12.3)	0.816
Gender (M/F)	4/29	9/28	0.230
BMI*	26.0 (3.7)	26.3 (3.4)	0.668
ASA grade*	2.0 (0.5)	2.0 (0.6)	0.886
Involved diseases			
OA	4	6	0.616
RA	29	31	
Preoperative Hb* (g/L)	131.2 (5.9)	132.1 (5.6)	0.514
Preoperative Hct* (%)	40.2 (1.5)	40.4 (2.7)	0.701
Preoperative serum level of albumin* (g/L)	43.0 (3.5)	42.9 (4.6)	0.947
Preoperative VAS pain score*	8.1 (0.9)	8.1 (1.0)	0.821
Preoperative HSS (points)*	38.7 (9.8)	38.2 (6.5)	0.739

*Abbreviations*: *BMI* body mass index, *OA* osteoarthritis, *RA* rheumatoid arthritis, *Hb* hemoglobin, *Hct* hematocrit, *ASA* American Society of Anesthesiologists, *VAS* visual analog scale, *HSS* Hospital for Special Surgery.

*The values are given as mean ± standard deviation.

Significant differences were observed between the groups in terms of drainage volume and estimated blood loss (Table 2). A mean decrease of 603 mL of estimated blood loss was found in Group A (*p* <0.001). As to the hidden blood loss and the highest changes of both Hb and Hct, priority was also revealed in Group A (Table 2).

**Table 2:** **Intraoperative and postoperative demographics**

**Demographic**	**Group A (** ***n*** **=33)**	**Group B (** ***n*** **=37)**	***p*** **value**
Duration of surgery* (min)	117.7 (17.1)	121.5 (15.7)	0.336
Drainage volume* (mL)	279.2 (78.0)	458.4 (96.4)	<0.001^‡^
Calculated blood loss* (mL)	1227.2 (196.1)	1830.2 (349.6)	<0.001^‡^
Hidden blood loss* (mL)	947.9 (200.9)	1371.9 (372.7)	<0.001^‡^
Interval days* (d)	3.4 (0.6)	3.6 (0.5)	0.345
Length of stays* (d)	12.4 (1.2)	13.2 (1.5)	0.012^‡^
The highest changes of Hb* (g/L)	37.7 (3.8)	47.2 (4.3)	<0.001^‡^
The highest changes of Hct* (%)	11.6 (1.4)	15.2 (1.6)	<0.001^‡^
HSS score at discharge* (points)	82.1 (5.7)	80.8 (6.8)	0.319

*Abbreviations*: *Hb* hemoglobin, *Hct* hematocrit, *HSS* Hospital for Special Surgery.

*The values are given as mean ± standard deviation.

^‡^Significantly different.

When transfusions were compared for surgeries performed with different blood managements, significantly different transfusions rates were found: mean in Group A was 9% (*n* =3/33), and in Group B was 32.4% (*n* =12/37) (*p* =0.021) (Table 2). As to the number of transfusions per patient, less transfusion was also observed in the patients of Group A (0.24 ± 0.83 IU VS 0.81 ± 1.28 IU, *p* =0.030) (Table 2).

### Nutritional status

**Figure 3:**
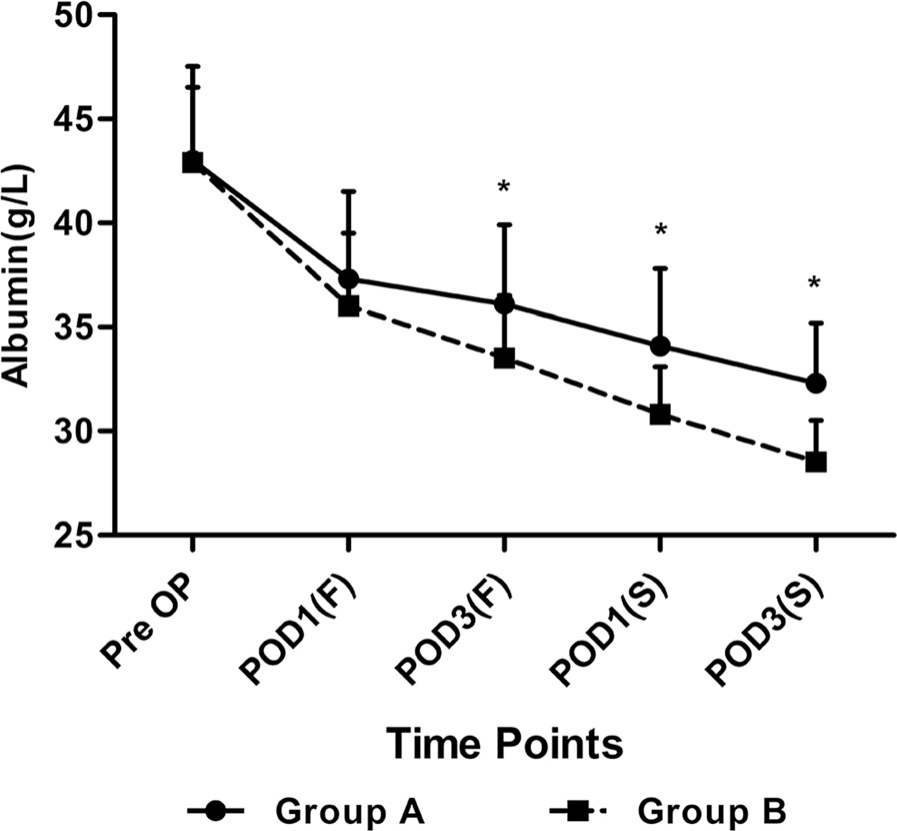
**The perioperative serum levels of albumin at each time point.** *Pre OP* preoperative, *POD1(F)* 1st day after the first surgery, *POD3(F)* 3rd day after the first surgery, *POD1(S)* 1st day after the second surgery, *POD3(S)* 3rd day after the second surgery. The asterisks indicate values that were significantly different between the groups.

The extent of the serum levels of albumin at different time points was listed as primary outcomes which can reflect the nutritional status of the patients. Significant differences were detected between the two groups on POD3(F) (36.1 ± 3.8 VS 33.5 ± 3.0, *p* =0.003), POD1(S) (34.1 ± 3.7 VS 30.8 ± 2.3, *p* <0.001) and POD2(S) (32.3 ± 2.9 VS 28.5 ± 2.0, *p* <0.001) (Figure 3).

### Pain, swelling, postoperative course, and hospital cost assessment

**Figure 4:**
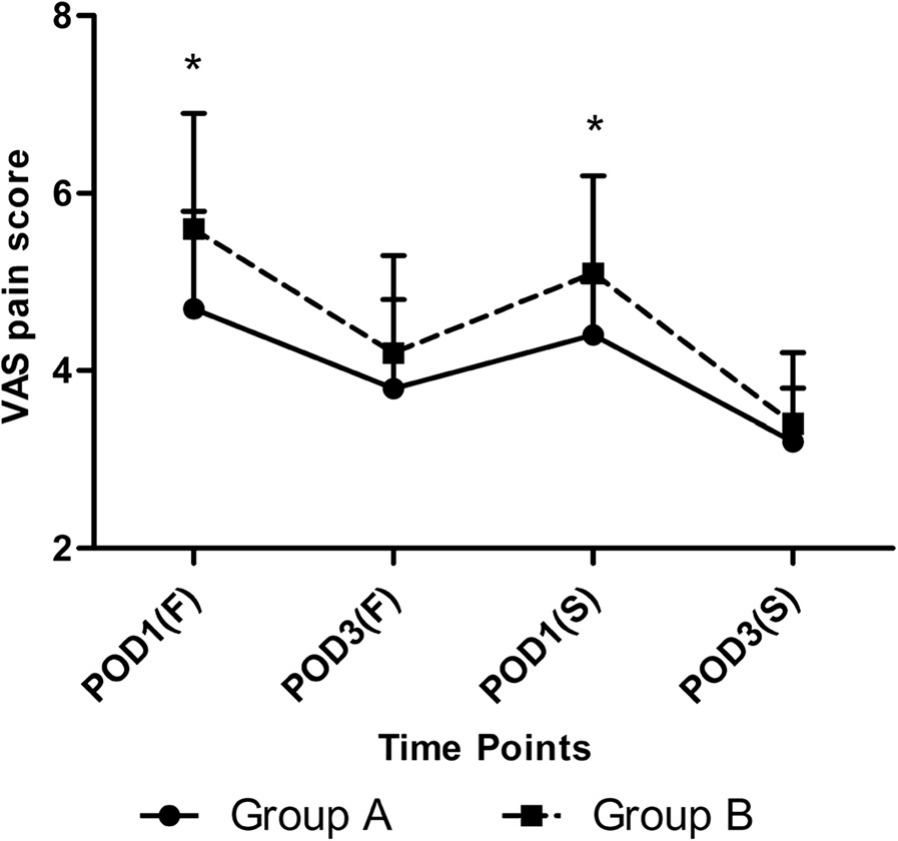
**The postoperative visual analog scale (VAS) scores at each time point.** *POD1(F)* 1st day after the first surgery, *POD3(F)* 3rd day after the first surgery, *POD1(S)* 1st day after the second surgery, *POD3(S)* 3rd day after the second surgery. The asterisks indicate values that were significantly different between the groups.

Postoperative pain decreased daily though there was a fluctuation between the POD3(F) and POD1(S). Group A had slightly less postoperative pain on POD1(F) (4.7 ± 1.1 VS 5.6 ± 1.3, *p* =0.001) and POD1(S) (4.4 ± 0.6 VS 5.1 ± 1.1, *p* =0.002) (Figure 4). The results of mean preoperative increment of both thigh girth and calf girth showed less severity of swelling on POD1 and POD3 (Table 3). The average length of hospital stay was 12.4 ± 1.2 days in Group A and 13.2 ± 1.5 days in Group B (*p* =0.012), while no significant differences were detected in terms of interval days (Table 2). The assessment of hospital costs showed there was an average increase of ¥1,638 per case in Group B.

**Table 3:** **Mean (SD) increments in circumferences of the leg over time for the two groups**

**Groups**	**Pre-operation (cm)**	**Preoperative increment** ^**†**^	
		**POD1**	**POD3**
Thigh girth^a^			
Group A	36.5 (3.8)	1.4 (2)	1.2 (1.8)
Group B	37.5 (3.1)	1.5 (1)	1.3 (0.8)
*p* value	0.246	0.011*	0.001*
Calf girth^b^			
Group A	29.9 (2.0)	0.7 (2)	0.5 (1.6)
Group B	30.3 (2.7)	0.9 (2)	0.6 (1.9)
*p* value	0.527	0.038*	0.010*

^a^Thigh girth: circumference at the thigh, 10 cm proximal portion form the top of the patella.

^b^Calf girth: the maximum circumference of the calf.

^†^Preoperative increment: increment of the circumference compared with that of the pre-operation.

*Statistically significant difference between the two groups: *p* <0.05.

### Functional assessment

The HSS score was used to assess patients’ knee function. Baseline demographics were similar between the two groups (Table 1). At the time of discharge, a substantial improvement was found in both groups: mean HSS scores increased to 82.1 (5.7) in Group A and to 80.8 (6.8) in Group B. Yet, there was no difference approaching significance between groups in the HSS scores at discharge, though Group A showed a little bit higher (Table 2).

### Complications

No DVT was found in any patients of the two groups, while intermuscular venous thrombosis was found in 5 patients of Group A and 3 patients of Group B.

## Discussion

With the increasing success of the TKA as an extremely effective intervention for end-staged knee diseases, how to improve the effectiveness and lower down the risks of complications has become the first concern of all the joint surgeons [1]. Among them, blood loss conservation is a major one. In this retrospective study, we have compared the outcomes of the newly introduced MBM with those of traditional one applied in the patients undergoing staged bilateral TKA in a single hospitalization period.

Due to its retrospective nature, this study has several limitations, including the non-randomized design, not allowing for complete control of the perioperative variables such as demographics and co-morbidities. In addition, the use of the different prostheses may contribute to some limitations. Despite these limitations, the strengths of our study were as follows: First, this study focused on a relatively large number of cases; second, the variables were reduced to a minimum because the patients were treated by the same surgical team, using the same familiar surgical technique. In addition, we were able to pinpoint the exact transfusion needs in TKA while the current literature on blood management in orthopedics tends to report contradictory statements with no definitive conclusions.

One of the important findings in this study is that the newly introduced MBM can effectively diminish the reduction of both Hb and Hct levels by 3 days post-operation. Less calculated blood loss and drainage volume were also found in Group A. As was reported previously [1], the lowest Hb and Hct levels always appear on the postoperative day 3, we believe by taking the newly introduced MBM, the perioperative blood loss in the patients undergoing staged bilateral TKA in a single hospitalization can be effectively reduced. A transfusion rate of 9% was obtained in Group A, while in Group B the transfusion rate was 32.4%. We believe that these findings provide a further proof about the non-pharmacological protocols—including a high protein diet, many surgical gestures, not only pharmacological protocols—hematinics, and TXA.

As an inhibitor of fibrinolysis, TXA has gained significant popularity in TKA. Many prospective randomized clinical trials and meta-analysis have reported that TXA can limit blood loss following unilateral TKA [8,9]. While the literatures in this field are abundant, the lack of studies related to bilateral TKA makes our study salient. Recently, Kelley et al. [10] has conducted a retrospective case–control study comparing blood loss after bilateral TKAs staged 3 days apart (2 doses of 1 g) versus placebo. Significantly less blood loss occurred in the TXA treatment group versus controls. Also fewer transfusions per patient and total drain output were found in the TXA group. These finds were quite similar to ours.

However, our TXA administration regimen differed from the protocols used in that study [10], as we administered TXA in a single intravenous dose of 15 mg/kg just before the inflation of the tourniquet. When we used tourniquet during the surgery, the blood loss of the surgical field was too little to be calculated, so we considered it unnecessary to use the TXA before the incision. Application of pneumatic tourniquet enhances fibrinolysis as a result of plasminogen activator release from the vascular endothelium, triggered by hypoxia or venous stasis. Starting an antifibrinolytic therapy before tourniquet could interfere with the natural defense mechanisms of the body against thrombus formation. As to the dose of TXA, it is still under debate [11-13]. According to a meta-analysis conducted by Cid et al. [11], the reduction in the risk of receiving a blood transfusion was independent of the total dose of TXA given. And some other studies [12] reported the low dose of TXA (15–35 mg/kg) was sufficient to lower down the blood transfusion requirement. Since the risk of thrombosis might increase with the growth of the dose of TXA, we used the dose of 15 mg/kg.

It was interesting to notice that the hidden blood loss in Group A was much less than that in Group B, which seems to be inconsistent with the previous study [14], which reported that the IV administration of TXA could only decrease external blood loss instead of the hidden blood loss after TKA. We contributed the phenomenon to the reasons as follows: First, the previous study focused on the unilateral TKA rather than the bilateral ones [14]. So, the reduction of the hidden blood loss might be too little to be detected. Second, as a result of using a high protein diet to the patients in Group A, the reduction of albumin was slowed down; this might accelerate the repair process of the tissue and result in the less hidden blood loss. What is more, a multimodal blood saving protocol was used in this study, other approaches, such as a high protein diet, surgical gestures, and hematinics might also have some impact on it [7,15].

With the decrease of the hidden blood loss in the joint, the benefit in reducing the joint swelling emerged [16]. According to Bergin et al.’s study [17], the blood loss and transfusion requirements could be regarded as a sign of tissue trauma. With less blood loss, the inflammation of the tissue was less severe. Thus, smaller increment of both thigh girth and calf girth was found on POD1 and POD3. Additionally, the less severity of the inflammation of the tissue means less pain, which was observed on POD1(F) and POD1(S). However, this kind of advantage disappeared on the POD3(F) and PDO3(S). We believed the standard postoperative pain control protocol might contribute to the phenomenon.

As for the length of hospital stays, we found a shorter length of stay was associated with the application of the newly introduced MBM. The possible reason why the length of stays is shorter in Group A is that the patients could be more aggressively mobilized. It is unnecessary for the patients to avoid physical therapy to receive transfusions. Instead it was better for them to participate in physical therapy, as they did not have symptoms of anemia. Thus, the patients in Group A could be discharged earlier.

Previously, Sepah et al. [18] and Ralley et al. [13] conducted several studies to assess the costs of using IV-administered TXA in TKA. In our study, we directly compared the costs related to the pharmacy, transfusions, and length of hospital stays to calculate the total direct costs. Although using the newly induced MBM could cause the rise of the pharmacy costs, this was more than an offset by the pharmacy costs associated with a shorter length of hospital stays. The saving was a total of ¥1,638 per case, which is quite a large sum of money for a Chinese family.

The theoretical risk of using TXA is thrombosis. A recent meta-analysis by Yang et al. [9] showed the use of TXA was not associated with increased perioperative complications. However, Yeager et al. [4] reported the patients who underwent bilateral TKA were at a higher risk of developing DVT or even PE. However, we did not see a significant increase in DVT in our study. The reasons might be as follows: First, in order to lower down the risk of DVT, we moved the usage of the LMWH 2 h ahead to the postoperative 6 h; second, a regular protocol of thrombosis prevention, including medical and physical approaches, was followed well in the patients of both groups even after the discharge; furthermore, early rehabilitation activities which can reduce the risk of thromboembolism were encouraged in the physical therapy process of our center. What is more, according to the study of Lee [19], Asians are less likely to develop DVT than Caucasians.

In conclusion, we found that the newly introduced MBM in staged bilateral TKA in a single hospitalization can reduce blood loss effectively as well as pain and knee joint swelling instead of leading to increased complications and result in significant cost savings.

## Abbreviations

TKA: Total knee arthroplasty
BMI: Body mass index
OA: Osteoarthritis
RA: Rheumatoid arthritis
Hb: Hemoglobin
Hct: Hematocrit
ASA: American Society of Anesthesiologists
VAS: Visual analog scale
HSS: Hospital for Special Surgery
POH1: Postoperative hour 1
POH1(F): 1st hour after the first surgery
POH1(S): 1st hour after the second surgery
POD1: Postoperative day 1
POD1(F): 1st day after the first surgery
POD1(S): 1st day after the second surgery
POD3: Postoperative day 3
POD3(F): 3rd day after the first surgery
POD3(S): 3rd day after the second surgery
